# The role of liver microenvironment in hepatic metastasis

**DOI:** 10.1186/s40169-019-0237-6

**Published:** 2019-07-01

**Authors:** Tovah Williamson, Nikhila Sultanpuram, Hossein Sendi

**Affiliations:** 1 Department of Radiation Oncology, UNC School of Medicine, Chapel Hill, NC USA; 2 Center for Nanotechnology in Drug Delivery, UNC School of Pharmacy, Chapel Hill, NC 27599 USA

**Keywords:** Cancer, Tumor, CTC, ECM, Metastatic

## Abstract

Metastasis is still poorly understood and thus further research must be conducted to provide insight into the driving factors. Novel research has revealed the significance of the microenvironment in the delegation of metastasis, expanding the field of cancer metastasis to cells and cell environments surrounding the migrated tumor cells. Research on hepatic metastasis is an ever-growing domain of this field, as several primary tumors can metastasize to the liver. The two features within the liver that promote metastasis—cellular and acellular—are found in the current interpretation of liver microenvironment. Novel findings of both are included in this review. Different hypotheses detailing the methods by which metastasis can occur must be included to understand the significance of the microenvironment, as well as a brief overview of the methods that can be used during research. This review aims to highlight the importance of liver microenvironment on the development or potential regression of hepatic metastasis through discussing both acellular and cellular components of liver microenvironment and their interaction with metastasis.

## Introduction

Organ microenvironments are an undeniable component for metastasis, enabling proliferation of cancer cells to organ systems external to the primary tumor. Migration of cancer cells requires compatibility with the destination, particularly with the microenvironment. Hepatic metastases can arise from the primary cancers in different locations within the human body. Both the acellular-such as extracellular matrix (ECM) proteins (i.e. collagen) and the cellular components of the liver such as Kupffer cells (KCs), hepatic stellate cells (HSCs), and liver sinusoidal endothelial cells (LSECs) contribute to the metastatic ability of tumors of different origins. Common incidences of liver metastasis occur in colorectal cancers, as these cells can take advantage of both the proximity (as a great amount of venous drainage is to the liver) and highly vascular nature of the liver [1]. A statistical analysis revealed that of 4399 patients with cancer, 41% of them experienced metastasis to the liver, marking it as the second greatest metastatic site, just behind nonspecific lymph node metastases [2].

The ECM is the major acellular component contributing to the liver microenvironment, and consequently the microenvironment of the tumor. The matrix of biological tissue serves as a framework by which cells organize, intertwined with the local vasculature. The ECM also contains domains that allow proteins to bind, which are important for cell–cell communication as well as specific function. ECM proteins can interact with growth factors, such as hepatocyte growth factor (HGF) which promotes cell migration and vascular endothelial growth factor (VEGF) which can enable metastasis progression through angiogenesis [3]. The major cellular components, such as Kupffer cells, liver sinusoidal endothelial cells, hepatocytes, etc. are also involved, primarily through communication with matrix and intracellular proteins, to promote metastasis.

Though the liver microenvironment can promote metastasis, the liver will initially respond in ways to react to inflammation or damage. This premetastatic niche can protect the liver, using cellular signaling to promote immune responses. Both cellular and acellular provide these dueling roles, such as KCs and cytokines, though eventually the prometastatic elements outweigh the opposition as metastasis occurs [4]. Table 1 summarizes major cellular and acellular components play role in hepatic metastasis identified in previous studies. The initial microenvironment can play opposing roles in the development of metastases, but once tumor growth occurs, that environment is subject to changes that ultimately support that growth.

**Table 1:** Major acellular contributors to metastasis

Name	Possible involvement	References
CEA	Attachment of CTC to fibronectin	[5, 6]
CXCL8 (IL-8)	Tumor proliferation	[8]
VEGF	Angiogenesis	[9]
MAPK	Promotion of EMT	[9]
TGF-β		
NF-κB		
PAD	Catalyze citrullination of proteins	[10, 11]
MMP-2	Increased protein turnover in ECM	[13]
Periostin		
MMP-9		
Type I collagen	Induce metastatic properties of surrounding environment	[14, 41]
Type IV collagen		[15]
Major cellular contributors to metastasis		
Kupffer cells, TAM	M1 to M2 transition circumvents immunogenic response	[4, 12, 16, 18, 42]
TAM	Immunosuppressive properties	[19, 25, 26]
TAN		
MDSC		
LSEC	Increase angiogenesis, induce EMT	[4]
HSC	Progenitors of CAF	[21, 22]

## Acellular components

The major acellular components that support or are involved in metastatic niche formation are as follows; the cell-adhesion molecule carcinoembryonic antigen (CEA) and other cell adhesion molecules (CAMs), CXC motif-chemokines (CXCLs), VEGF, MAPK, and NF-κB, Citrullinated proteins/PAD, Spermine pullulan, matrix metalloproteinases, and collagen proteins (Table 1).

### Carcinoembryonic antigen

Abdul-Wahid et al. reported that the CEA, which can be expressed on the surface of colon circulating tumor cells (CTCs), contributes to the subsequent attachment to fibronectin of the liver ECM, resulting in increased levels of metastasis [5]. This is supported in the review by Rizeq et al., which describes in further detail of human CEA and other cell adhesion molecules, showing its role in cancer progression [6]. It reiterates the ability of these molecules to increase the binding of fibronectin in the ECM to cancer cells, as well as participating in more direct proliferative measures such as apoptosis, all of which mark the beginning of metastasis [6].

### CXC motif-chemokines

It is known that CXC motif-chemokines (CXCL) 1, 2, 3, and 5 contribute to colorectal carcinoma, but novel exploration expanded this understanding to metastasis to the liver. Knock-down of Interleukin-8 (IL-8, or CXCL8), which can be produced by macrophages, was shown to inhibit tumor growth in colorectal liver metastasis. This was associated with the reduction in cell proliferation and viability in samples deficient in CXCL8 [7].

### VEGF, MAPK, and NF-κB

VEGF expression levels were also decreased during knock-down of CXCL8, suggesting a mechanism for the decreased proliferation [8]. VEGF, along with MAP Kinase (MAPK) and NF-κB, were reported to contribute to liver metastasis of breast cancer [9]. A study identified different genes associated with patients that developed liver metastases from a primary breast tumor, and found that these signaling pathways were highly conserved among patients, since VEGF promotes angiogenesis (required for tumor growth and expansion), MAPK promotes epithelial-to-mesenchymal transition (EMT) associated with TGF-β, and NF-κB is a regulatory transcription factor for the immune response and is associated with EMT as well [10].

### Citrullinated proteins and PAD

Recently, ECM proteins were shown to have a greater incidence of citrullinated proteins, catalyzed by peptidylarginine deiminase proteins (PAD), which is specifically seen in liver metastases. Citrullination occurs on the arginine, producing a neutrally charged product. It was suggested in earlier studies that citrullination of certain proteins, which are prevalent and detectable in tumor cells, contribute to the progression of the tumor [11]. A more recent study focused on PAD4 and colorectal cancer metastasis to the liver, showing that the downregulation of PAD4 reduced metastatic growth, suggesting the possibility of citrullination as a contributor to metastasis [11].

### Spermine pullulan

Polysaccharide spermine modified pullulan (SP), which is produced by the fungus *Aureobasidium pullulans*, can be utilized to polarize macrophages towards M1, which can ultimately inhibit metastasis by increasing inflammation [12].

### Matrix metalloproteinases

In an experiment involving the treatment of murine models with chemotherapy, periostin and matrix-metalloproteinase 2 (MMP-2) were increased when murine models were treated with the drug cisplatin. Therefore, they concluded that cisplatin may induce liver metastasis of murine melanoma cells via increase in MMPs, which in turn can manipulate the extracellular environment by protein turnover [13]. The study also looked at how treatment of the chemotherapy drug vincristine could aid in the metastasis of human neuroblastoma to liver, via an increase in MMP-9 expression [13]. The results of this study suggest that the microenvironment interactions with chemotherapy can not only increase understanding of metastasis, but provide valuable information regarding treatment expectations.

### Collagen proteins

Observational data show colorectal liver metastasis (CRLM) patients have elevated levels of type I collagen in the urine and plasma [14]. This report prompted further exploration of other ECM collagens [14]. Elevated levels for CRLM patients compared to controls were analyzed with a P value < 0.0001. Collagen peptides and alpha chains were identified, and the majority were upregulated in CRLM patients. This study underwent extensive data analysis, finding possible markers as well as drivers for metastasis. It was additionally hypothesized that abnormal ECM protein synthesis and degradation occurred, as it was determined that collagen turnover related proteins were upregulated in CRLM patients as well [14]. Data analysis on breast cancer metastasis determined that the addition of type I collagen altered the metastatic properties of tumor epithelia, allowing for a greater incidence of lung metastasis as well as an increase in the number of CTCs [13]. Though different mechanisms are involved in metastasis to the lung as opposed to metastasis to the liver, this provides an alternate view, accentuating the significance of collagen type I. Collagen type I was reviewed as a possible instigator of metastasis in these particular studies, and other experiments looked at metastasis occurring with other collagen types. A study on collagen IV and liver metastasis revealed the significance of type IV collagen expression, showing how upregulation of collagen IV is a possible driver of metastasis, and that downregulating this ECM protein reduces metastasis [15]. These articles collectively address the importance of matrix components in relation to the metastatic capacity of the tumor.

## Cellular components

**Fig. 1:**
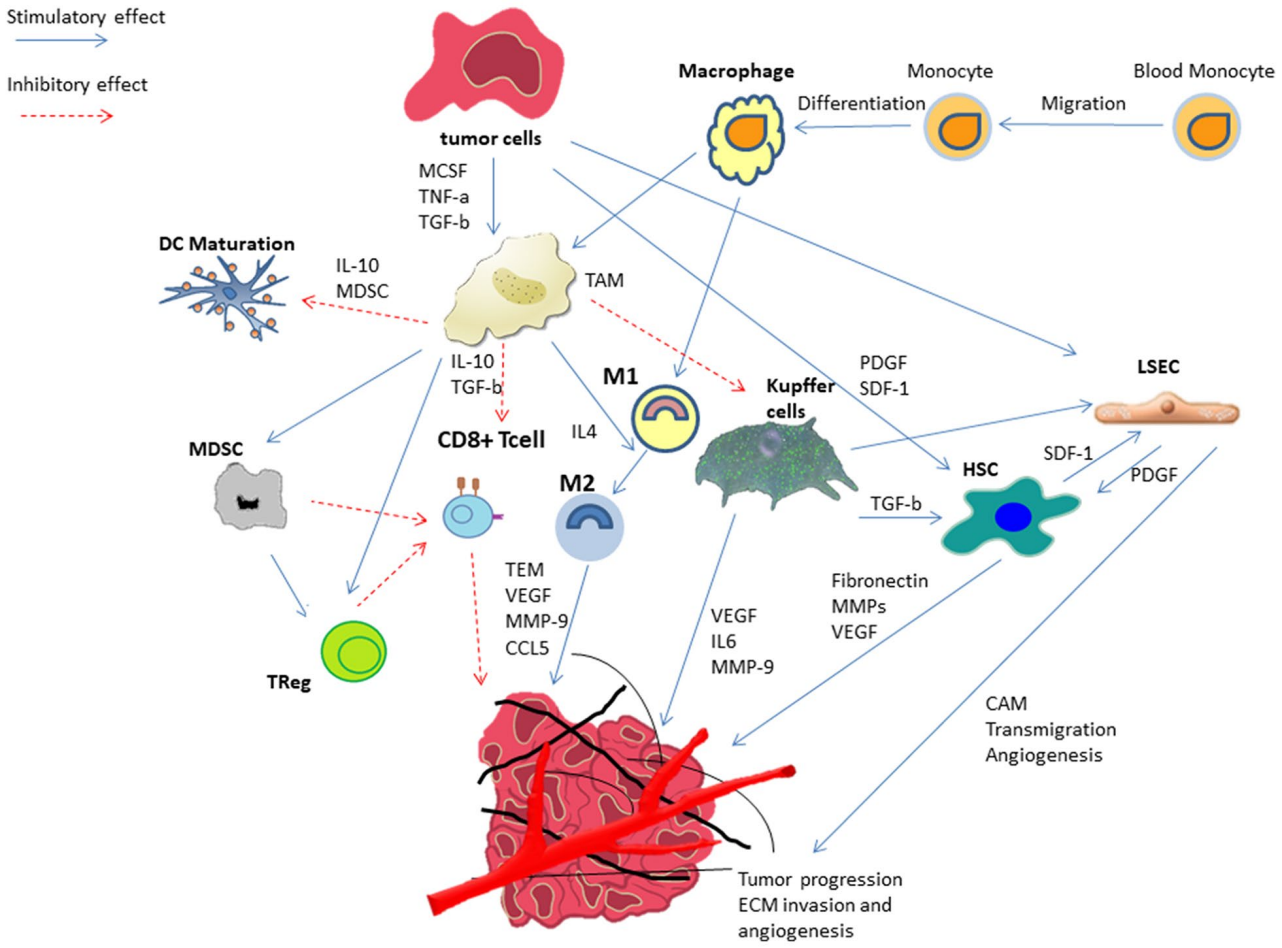
Cellular factors playing role in formation and development of metastatic niche within liver microenvironment. This figure depicts the schematic representation of the major cellular components involved in a multistep process in the formation and development of the metastatic niches in the liver microenvironment. (*TEM* transendothelial migration, *MMP* metalloproteinases, *CCL5* chemokine ligand 5)

Cellular components of the liver are as follows: bone-marrow-derived macrophages such as tumor-associated macrophages (TAMs), Kupffer cells (KCs), liver sinusoidal endothelial cells (LSECs), immune cells like tumor-associated neutrophils (TANs), myeloid-derived suppressor cells (MDSC), fibroblasts, hepatocytes, and hepatic stellate cells (HSCs) [4] (Fig. 1, Table 1).

### Macrophages and Kupffer cells

Macrophages can either be resident like KCs, or monocyte-derived, both of which can undergo M1 to M2 repolarization [16]. This transition circumvents the pro-immunogenic response and anti-tumorigenic qualities of these immune cells, assisting tumor growth. TAMs which take on the M2 phenotype, will prevent immunogenic and inflammatory responses and enhance cancer progression [12]. In addition, M2 phenotype can combat chemotherapy, thus preventing macrophage M2 polarization (thereby inducing M1 polarization) is a plausible area for treatment. Both cell types can contribute to metastasis [4], however some studies have suggested a larger role for KCs in their prevention of metastasis. A study by Kimura et al. reports lectin receptors (Dectins 1–3) can suppress tumor growth by promoting KCs and natural killer cells (NK cells) [17]. KCs are able to promote a viable environment for cancer cells, as they can indirectly promote the production of fibronectin [18]. However, KCs are a double edged sword, known for anti-metastatic properties as well [18]. Since the relationship of KCs to cancer progression is complex, the downstream effects of these cells needs to be further identified to enable probable therapeutic outcomes.

### Sinusoidal endothelial cells

Liver sinusoidal endothelial cells (LSECs) can provide both anti-metastatic and pro-metastatic functions. For example, LSECs can undergo apoptosis or release toxins to aid in elimination of tumors at the site of metastasis, or these cells can increase angiogenesis or EMT to promote metastasis [4].

### Immune cells

Different immune cells, such as dendritic cells, tumor-associated neutrophils (TAN), myeloid-derived suppressor cells (MDSC), and TAMs are all associated with different cytokines/chemokines or other factors that contribute to metastasis, summarized in Table 1 of the review by Smith and Kang [19]. Many of these factors are acellular components, though all acellular components must be considered within the context of the cellular origin or target. This is particularly important when discussing the role of immune cells, which frequently must intercommunicate with cytokines/chemokines.

### Fibroblasts

As mentioned, chemokines can contribute to metastasis by immune system suppression. The behavior of macrophages and other immune cells towards cancer is dependent on these cell-signal molecules. SDF-1 (CXCL12), or stromal cell derived factor, is known to allow for TAM movement, and can be secreted directly from cancer cells [20]. SDF-1 production is the result of activation of HSCs, which are thought to be progenitors to α-smooth muscle actin (α-SMA)-positive myofibroblasts [21] This phenotype is usually a marker for cancer-associated fibroblasts, which are tumor-activated forms of fibroblasts that secrete matrix proteins such as collagen, and can enhance the metastatic properties of the tumor [22]. The interaction between SDF-1 and CXCR4 can be interrupted as well, and targeting CXCR4 has shown to reduce metastasis, as SDF-1/CXCR4 expression and binding induces migration of cancer cells [22, 23]. Treatment with a CXCR4 antagonist was able to reduce metastasis in endometrial cancer (induced by cancer-associated fibroblasts) [22]. A recent study showed that the addition of a CXCR4 antagonist also disturbs metastasis of colorectal cancer to the liver, and connected certain HSC-derived factors to the increased expression of CXCR4 [24]. Hypoxia-inducing factor-1(HIF-1), can stimulate the transition of macrophages from M1 to M2 [20]. The conversion back to M1 can be induced through signaling molecules such as tumor necrosis factor alpha (TNF-α) and lipopolysaccharide (LPS) [4], as well as  SP (earlier stated); however, this could prolong the inflammatory response systemically. Insulin-like growth factor 1 (IGF-1) has been shown to induce metastasis and the signaling pathways involved in the promotion of EMT. Therefore, depletion of IGF-1 and prevented polarization of TAN to their pro-metastatic (N2) phenotype [25, 26].

## Cancer stem cells and tumor microenvironment

Cancer stem cells (CSC) are thought to be a portion of the population of a tumor, and can be differentiated based on their surroundings. The tumor microenvironment is crucial to this differentiation. CSCs can interact with both acellular and cellular components of the microenvironment. The review by Lau et al. describes the connections between CSC pathways and ECM changes, hypoxia, growth factors, etc., which all contribute to metastasis [27]. The development of CSCs and their role in metastasis needs to be studied further, though many hypotheses have been developed to explain this relationship. One particular hypothesis in regards to the relationship between CSCs and the EMT, which is currently not completely understood, involves the requirement of the microenvironment to provide signals and structure to which the CSCs can react [28, 29]. A specific example involves the migration of pancreatic adenocarcinoma to the liver, in which Knaack et al. studied in vitro, manipulating CSCs as well as the microenvironment by incorporating HSCs and hepatic myofibroblasts (HMF) to mimic physiological and inflammatory conditions. The data expressed the importance of HSCs and HMF in the formation of disseminated pancreatic ductal epithelial cell (PDEC) colonies, showing a marked relationship between liver microenvironment and pancreatic CTCs [30]. In addition, these colonies expressed a greater amount of Nestin, a CSC-marker, than their counterparts, which reveals the increased metastatic capabilities of these cells [30].

## The role of exosome in establishing liver metastasis

Recent studies have shown that pancreatic ductal adenocarcinoma (PDAC)-derived exosomes stimulate liver pre-metastatic niche formation (PMN). Costa-Vista et al. showed that uptake of PDAC-derived exosomes by Kupffer cells caused TGF-β secretion and upregulation of fibronectin production by HSCs [31]. They found that macrophage migration inhibitory factor (MIF) was highly expressed in PDAC-derived exosomes, and its blockade impeded liver pre-metastatic niche formation and metastasis [31]. This group also showed that exosomes from pancreatic cancer cell lines that metastasize selectively to the liver fused preferentially with KCs in the liver. Interestingly, Costa-Silva et al. found that exosomal MIF upregulation in mice with pretumoral pancreatic lesions, and high levels of exosomal MIF in plasma were also identified in patients with stage I PDAC. These results suggest that these exosomes could be formed at very early stages of cancer development [31].

## Epithelial-to-mesenchymal transition and circulating tumor cells

It is assumed that EMT is a primary method of metastasis, as the transition to mesenchymal cells allows for migration to the bloodstream, which acts as a highway for tumor cells. The reverse, mesenchymal-to-epithelial transition (MET), can halt tumor cells at a certain location. Individual cells detected in patient serum are known as circulating or disseminated tumor cells (DTCs), and it is thought that EMT plays a role in tumor cell dissemination [32]. The path of a CTC following EMT for liver metastasis is the infiltration of that cell into the sinusoids and ultimately extravasation, assuming survival of the CTC [33]. Monitoring CTCs in serum is difficult, as there are approximately 5 cells per 10 mL of blood [32]. However, discussion of CTCs in the formulation of a mechanism for cancer metastasis is still important. Interestingly, CTCs can differ from the primary tumor genetically, which increase the difficulty in detection, as DNA analysis can be used as a method of characterization [32]. Without proper characterization, the interaction between CTCs and the liver microenvironment is concealed.

## Methods for studying microenvironment and metastasis

The tumor and organ microenvironment is not as easily manipulated in laboratory settings; in vitro is not primarily involved in the components surrounding individual cells. This barrier prompted the exploration into new methods of visualization and manipulation. Both two-dimensional (2D) and three-dimensional (3D) models can be utilized, and have their respective advantages and disadvantages [34]. In vitro 2D models are relatively limited in the study of metastasis, especially in the context of the surrounding microenvironment, thus 3D models are typically used. However, in vivo models are expensive and inefficient, thus prompting exploration of different methods of cancer metastasis visualization that allow for environmental control. Organoids, aptly named to indicate its three-dimensional nature, are clusters of cells that represent a fraction of a particular tissue environment [35]. Use of organoids to represent hepatic tissue contain a greater representation of the liver proteins and genetic components than two-dimensional structures, and can serve as an advantageous method to study liver microenvironment [36]. Organoids were generated in a study of colorectal cancer liver metastasis, and this supported the use of organoids to study metastasis as certain biomarkers were retained, supplemental growth was established, and drug screening was successful [37]. Decellularization of tissue is a relatively new method of in vitro research, and can be used to study how the microenvironment of a particular organ interacts with different cell lines [38]. The decellularized tissue, known colloquially as biomatrix, can be pulverized and plated, which increases trial efficiency, or the structure of the organ can be retained, keeping this aspect of the microenvironment constant during experimentation [39]. In a study was done by Tian et al. in our lab, an ex vivo engineered metastatic model was developed on lung and liver biomatrix which can be used as new in vivo model for tissue-specific metastasis [40]. The review by Clark et al. discusses ex vivo systems, which can mimic in vivo and avoid certain limitations of murine models [33].

## Conclusions

Ultimately, the microenvironment of the liver is crucial to the development of hepatic metastases. Different growth factors, cell types, posttranslational modifications, signaling molecules, etc. are all necessary to understand, including their interactions, when deciphering the elements of metastatic outgrowth. The accumulation of observational and experimental studies contribute to the knowledge required to battle cancer metastasis, and the methods to understand this metastasis in laboratory settings continues to progress.

## Abbreviations

ECM: extracellular matrix
KCs: Kupffer cells
HSCs: hepatic stellate cells
LSEC: liver sinusoidal endothelial cells
HGF: hepatocyte growth factor
VEGF: vascular endothelial growth factor
CEA: carcinoembryonic antigen
CTC: circulating tumor cells
CEACAM6: carcinoembryonic antigen-related cell adhesion molecule 6
CXCL: CXC ligand
MAPK: mitogen-activated protein kinase
NF**-**κB: nuclear factor kappa-light-chain-enhancer of activated B cells
TGF-β: transforming growth factor beta
EMT: epithelial-to-mesenchymal transition
PAD: peptidylarginine deaminase
SP: spermine pullulan
MMP: matrix metalloproteinase
CRLM: colorectal liver metastasis
TAM: tumor-associated macrophage
NK cells: natural killer cells
TAN: tumor-associated neutrophils
MDSC: myeloid-derived suppressor cells
SDF: stromal cell-derived factor
HSC: hepatic stellate cell
HIF-1: hypoxia-inducing factor-1
TNF-α: tumor necrosis factor alpha
IGF-1: insulin-like growth factor -1
CSC: cancer stem cells
HMF: hepatic myofibroblasts
PDAC: pancreatic ductal adenocarcinoma
PDEC: pancreatic ductal epithelial cell
MET: mesenchymal-to-epithelial transition
DTC: disseminated tumor cells
2D: two-dimensional
3D: three-dimensional
